# Antimicrobial Resistance Profiles of Bacteria Isolated from the Nasal Cavity of Camels in Samburu, Nakuru, and Isiolo Counties of Kenya

**DOI:** 10.1155/2017/1216283

**Published:** 2017-09-24

**Authors:** J. M. Mutua, C. G. Gitao, L. C. Bebora, F. K. Mutua

**Affiliations:** Department of Veterinary Pathology, Microbiology and Parasitology, University of Nairobi, P.O. Box 29053-00625, Nairobi, Kenya

## Abstract

This study was designed to determine antimicrobial resistance profiles of bacteria isolated from the nasal cavity of healthy camels. A total of 255 nasal samples (swabs) were collected in Isiolo, Samburu, and Nakuru counties, Kenya, from which 404 bacterial isolates belonging to various genera and species were recovered. The bacterial isolates included* Bacillus* (39.60%), coagulase-negative* Staphylococcus* (29.95%),* Streptococcus* species other than* Streptococcus agalactiae* (25.74%), coagulase-positive* Staphylococcus* (3.96%), and* Streptococcus agalactiae* (0.74%). Isolates were most susceptible to Gentamicin (95.8%), followed by Tetracycline (90.5%), Kanamycin and Chloramphenicol (each at 85.3%), Sulphamethoxazole (84.2%), Co-trimoxazole (82.1%), Ampicillin (78.9%), and finally Streptomycin (76.8%). This translated to low resistance levels. Multidrug resistance was also reported in 30.5% of the isolates tested. Even though the antibiotic resistance demonstrated in this study is low, the observation is significant, since the few resistant normal flora could be harboring resistance genes which can be transferred to pathogenic bacteria within the animal, to other animals' bacteria and, most seriously, to human pathogens.

## 1. Introduction

Among the animals reared in the arid and semiarid areas of Kenya, camels are most suited to the harsh environmental conditions which are a characteristic of these areas. They play an important role in support of livelihood and culture of pastoral communities in the country [1]. Despite the benefits associated with camels, the animals face challenges in their natural habitat which include diseases, drought, inadequate veterinary services, and little attention by government agencies [2–4]. Respiratory infection is considered to be one of the emerging diseases causing considerable loss of camel productivity and death [5].

Isolation of bacteria from the respiratory system of both healthy and diseased camels has been reported by a number of researchers; Arora and Kalra [6] reported isolation of* Klebsiella pneumoniae* and diplococci from camel pneumonic lungs in India. Chauahan et al. [7] reported eight genera of bacteria from nasal swabs taken from apparently healthy camels. A study by Shigidi [8] in Somalia reported isolation of six genera of bacteria in the upper respiratory tract. Al-Doughaym et al. [9] reported 9 genera of bacteria from the respiratory tract of diseased and healthy camels in Saudi Arabia. Azizollah et al. [10] identified different genera of bacteria from samples collected before and after slaughter from the respiratory system of healthy camels in an abattoir in Iran. Other countries where the normal respiratory bacteria of camels have been studied include Nigeria [11], Ethiopia [12], and Somalia (Mogadishu) [13]. Despite these observations, studies on bacterial flora of apparently healthy camels are rare globally [5] with none reported in Kenya, although pastoralists in Kenya report high incidences of camel respiratory diseases. Camel respiratory infection was the most reported disease in northern Kenya after swollen gland syndrome [14].

Noting that respiratory infection is one of the emerging diseases of camels [5], studies on normal nasal flora of camels may uncover a possible source of bacteria for the infections. This is because, although under normal circumstances, concentration of the resident bacteria in the nostrils is maintained at a particular level; in cases of stress of any kind, the mucociliary and clearance mechanism of the respiratory system is suppressed, allowing for multiplication of the commensal bacteria which may then result in an abrupt shift from normal flora to pathogenic bacteria [15].

Antibiotics are commonly used for treatment and prevention of diseases as well as growth promotion in domestic animals [16, 17]. Overuse, underuse, and misuse of antibiotics have led to increased development of antibiotic resistance in the world [18]. This imprudent use and mismanagement of antibiotics in agricultural and livestock practices have increased incidences of multiple drug-resistant bacteria inhabiting domesticated animals [18, 19], a situation which is worrying, since it translates to drug resistance to human bacteria [20], more importantly the pathogenic ones. This is because it is estimated that 60% of bacteria that are pathogenic to humans are from animals [21], the major problem being that the same drugs/medicines are used in both animals and humans for treatment and prophylaxis.

Kenya is experiencing antibiotic resistance with high resistance rates being reported in microorganisms causing respiratory, enteric, and hospital-acquired infections [22]. Overuse and misuse of antibiotics, underuse, inappropriate dosing, poor quality antibiotics, and lack of restriction to selling and distribution of antibiotics (where sometimes antibiotics are sold at bus stops by hawkers) have greatly contributed to the development of antibiotic resistance [23] in Kenya. Difficulty in fixing this menace in the country has been attributed to lack of adequate regulatory authority and inadequate regulatory resources to enforce key policies that have been put in place [22].

A few studies on antibiotic resistance in Kenya in domestic animals have been documented; for example, bacterial isolates from chicken and swine have been reported to be resistant to Tetracycline and Sulphonamides. Also other bacteria isolated from pork tissues have demonstrated resistance to Ampicillin, Streptomycin, Tetracycline, and Chloramphenicol [22].

The existence of bacteria in the nasal cavities of apparently healthy camels in Kenya has not been documented previously. The sensitivity of these bacteria to commonly used antibiotics is also not known, a fact that cannot be ignored as antibiotic use not only targets pathogenic bacteria but also exerts selective pressure on normal flora (bacteria), leading to maintenance of antibiotic resistance in these bacteria [24]. This study intended to fill in these gaps, especially when considering the possibility that the resistant genes may be carried on plasmids, which have been demonstrated to be easily transmitted between bacteria by other researchers [25–28]. Thus, antibiotic-resistant normal flora can easily transfer antibiotic resistance to otherwise susceptible pathogens, posing serious problems in the treatment of camel respiratory infections, let alone the risk of transfer of resistance genes to human pathogens.

## 2. Materials and Methods

### 2.1. Study Areas and Sample Collection

Samburu, Isiolo, and Nakuru counties were selected for the study, specifically Naimaralal and Opiroi locations of Samburu, Burat and Isiolo west locations (Isiolo), and Gilgil area of Nakuru.

The areas were purposively selected as treatment of animal diseases in the pastoral communities of Kenya is mostly done without the benefit of laboratory testing as laboratories are almost nonexisting or dormant in most of these areas. This could contribute to the spread of antibiotic resistance due to misuse of commonly used antibiotics [29].

A total of 255 nasal swab samples were collected using the method described by Mohamed et al. [30]. Briefly, gloved hands were used to clean the external nares of camels before disinfecting them with 70% alcohol. Swabbing was done by introducing sterile cotton swabs directly into the nasal cavities and rubbing them smoothly against the mucosa in a circular motion. The swabs were then placed into bijoux bottles containing Stuart transport media (Oxoid Ltd., Hampshire, England), wrapped with air-tight polythene bags, put in cool boxes, and transported to Department of Veterinary Pathology, Microbiology and Parasitology, University of Nairobi, Upper Kabete, for processing.

### 2.2. Bacterial Isolation and Identification

This was done according to standard procedures [31, 32]. Nasal swabs were streaked onto both blood agar containing 5% bovine blood and MacConkey agar using a sterile inoculating loop. The agar plates were incubated aerobically at 37°C for 24 hours. After incubation, each different colony was examined macroscopically to note colonial morphology, presence or absence of hemolysis, and/or pigment production. Primary identification test involved Gram staining according to procedure described by Forbes et al. [33] and Bebora et al. [34] to test for reaction, cellular morphology, and spore formation. Biochemical tests were additionally done; they included catalase, coagulase, CAMP, and gelatin liquefaction tests.

### 2.3. Antibiotic Susceptibility Testing

Antibiotic susceptibility testing was done using disk diffusion method as described by Clinical and Laboratory Standards Institute [35]. 95 isolates were randomly selected for convenience from all bacteria isolated from the three counties. The isolates were tested for susceptibility to commonly used antibiotics including Ampicillin, Tetracycline, Streptomycin, Co-trimoxazole, Kanamycin, Gentamicin, Sulphamethoxazole, and Chloramphenicol (HiMedia).

Isolates were grown on blood agar for 24 hours; 5 colonies were picked from each plate and suspended in 5 ml of sterile normal saline which was then adjusted to a density approximately equal to McFarland Opacity Standard number 0.5. A dry sterile cotton swab was then placed inside the suspension; excess liquid from the swab was expressed against the wall of the tube and the swab used to spread the bacterial suspension evenly on the surface of Mueller-Hinton agar in order to get confluent growth. Antibiotic disks were then placed on the surface of the inoculum and incubated for 18–24 hours. Zones of inhibition were measured to the nearest millimeter and interpretation as to whether the bacterium was resistant or susceptible to the particular antibiotic was done according to specifications defined by Clinical and Laboratory Standards Institute (CLSI 2006).

## 3. Results

245/255 samples (96%) showed positive bacterial growth yielding different genera and species of bacteria. 404 isolates (all Gram-positive) were isolated from the 245 positive samples.

Isolated bacteria included* Bacillus* at 39.6% (160/404), coagulase-negative* Staphylococcus* at 29.95% (121/404),* Streptococcus* species other than* Streptococcus agalactiae* at 25.74% (104/404), and coagulase-positive* Staphylococcus* at 3.96% (16/404) and* Streptococcus agalactiae* was the least isolated at 0.74% (3/404). Organisms belonging to the genus* Bacillus'* species were the most frequently isolated in Isiolo at 56.07% (60/107), followed by Samburu at 40.85% (67/164) and Nakuru at 24.81% (33/133). Coagulase-negative* Staphylococcus* was most frequently isolated from Nakuru at 36.84% (49/133), followed by Samburu at 29.27% (48/164) and Isiolo at 22.43% (24/107).* Streptococcus* species other than* Streptococcus agalactiae* were the highest isolated from Nakuru at 35.34% (47/133), followed by Samburu at 21.95% (36/164) and Isiolo at 19.63% (21/107). Coagulase-positive* Staphylococcus (Staphylococcus aureus)* was the highest isolated from Samburu at 6.1% (10/164), followed by Nakuru at 3% (4/133) and Isiolo at 1.87% (2/107).* Streptococcus agalactiae* was isolated from Samburu at 1.83% (3/164); none was isolated from both Nakuru and Isiolo counties (Table 1).

**Figure 1:**
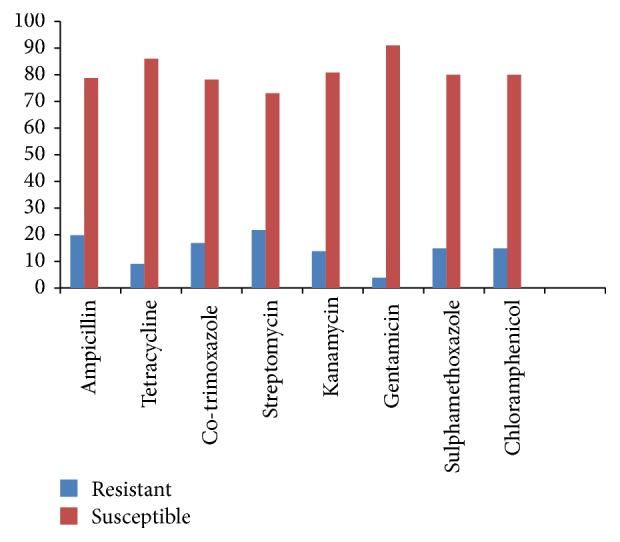
Antibiotic susceptibility profiles of the tested isolates: *n* = 95.

From the antibiotic susceptibility testing, the tested bacteria were highly susceptible to all the tested antibiotics as follows: Gentamyiin at 95.8% (91/95), Tetracycline at 90.5% (86/95), Kanamycin and Chloramphenicol at 85.3% (81/95), Sulphamethoxazole 84.2% (80/95), Co-trimoxazole at 82.1% (78/95), Ampicillin at 78.9% (75/95), and Streptomycin at 76.8% (73/95) (Figure 1). Thus, they showed minimal resistance to the antibiotics.

Although in this current study most isolates showed resistance to one antibiotic, some of the bacterial isolates in different genera and species showed multidrug resistance (resistance to more than one antibiotic), with respect to the antibiotics used. Generally, out of the 95 isolates tested, 29 (30.5%) showed resistance to more than one antibiotic used in this study.

## 4. Discussion

Camel keeping in Kenya, including the study areas, is commonly kept under traditional pastoral production system which is characterized by low production inputs and herd/household mobility which is necessitated by search of pasture, water, mineral licks, and community feuds [1]. Vaccination programs and treatment and control of diseases in cattle and other livestock exist but there are almost none for camels. Treatment of diseases is often done without benefit of laboratory examination as laboratories almost do not exist in these areas [29], which leads to misuse of antibiotics. Treatment is also mostly done by camel owners who have little or no knowledge of the diseases and the drugs they use; this has also led to spread of antibiotic resistance.

This study shows that diversity of bacterial species can be found in the nasal cavity of apparently healthy camels. The organisms could have reached the nasal cavity through inhalation, direct or indirect contact, or during drinking. However, the normal flora in apparently healthy camels can be altered by several factors such as bad sanitation, stress due to transportation, sudden change in feed, low herd health status, and immunosuppression. This could end up lowering the resistance of the respiratory system to infection [36]; thus, the existing nasal organisms could cause infections in the nasal cavity or end up finding their way down the system and eventually cause pathology in the lower respiratory system of the respective camels [37].

Isolation of diverse bacteria in this study has been supported by other authors who demonstrated presence of diverse bacterial species in the nasal tract of the apparently healthy camels [38] from nose, trachea, tonsils, and lungs of apparently healthy camels [10] and from lungs of apparently healthy and diseased lungs [39]. Al-Doughaym et al. [9] isolated* Staphylococcus aureus*,* Corynebacterium pyogenes*, coagulase-negative* Staphylococcus*,* Bacillus* species*, Streptococcus pyogenes, diphtheroids, E. coli, Klebsiella pneumoniae,* and* Diplococcus pneumoniae* from nasal swabs collected from camels in Sudan.

Generally, in this study, most of the isolates were sensitive to the antibiotics used. The susceptibility percentages of the organisms in the descending order were as follows: Gentamicin at 95.8% (91/95), Tetracycline at 90.5% (86/95), Kanamycin at 85.3% (81/95), Sulphamethoxazole and Chloramphenicol at 84.2% (80/95), Co-trimoxazole at 82.1% (78/95), Ampicillin at 78.9% (75/95), and finally Streptomycin at 76.8% (73/95). Gitao et al. [29] reported mastitic isolates from camel milk to be more susceptible to Gentamicin and Tetracycline. Most of* Staphylococcus aureus* and* Streptococcus agalactiae* were also reported to show marked resistance to Co-trimoxazole, Sulphamethoxazole, and Ampicillin [29]. A variety of studies in the last two decades have shown resistance to the commonly used antibiotics to be increasing including Co-trimoxazole, Ampicillin, Tetracycline, and Chloramphenicol [40, 41]. Similar results were also reported by Muna et al. [36], where bacteria isolated from camels suffering from pneumonia were susceptible to Gentamicin.

In this study,* Staphylococcus aureus* had the highest susceptibility to Gentamicin, Chloramphenicol, and Kanamycin followed by Tetracycline and Sulphamethoxazole, followed by Ampicillin, followed by Co-trimoxazole and Streptomycin. These results agreed to some extent with those of Abdulsalam [38]; he found* Staphylococcus aureus* to be highly sensitive to Ampicillin, Doxycycline, Streptomycin, Gentamicin, and Neomycin. A study by Gitao et al. [29] reported* Staphylococcus aureus* from mastitic milk in camels to be resistant to Ampicillin, Co-trimoxazole, and Sulphamethoxazole.

The susceptibility of coagulase-negative* Staphylococcus* in this study was least in Ampicillin and Tetracycline; a study by Al-Thani and Al-Ali [42] reported* Staphylococcus* species to be resistant to Tetracycline, Penicillin, and Ampicillin in different Qatari farms. However, the same study reported the organisms being very susceptible to Cephalothin, Norfloxacin, and Co-trimoxazole.

*Streptococcus* organisms had the highest susceptibility to Tetracycline followed by Ampicillin; they were also sensitive to Chloramphenicol and Gentamicin, followed by Sulphamethoxazole and then Co-trimoxazole, and they were least susceptible to Streptomycin and Kanamycin. This was in agreement with results obtained by Abdulsalam [38], where* Streptococcus* isolates were sensitive to Ampicillin and Doxycycline. However, Abdulsalam [38] reported* Streptococcus* organisms that were highly susceptible to Streptomycin at 100%.

Overuse and improper use of antibiotics have contributed to development of multidrug resistance in bacteria [18, 19]. In this study, a variety of genera and species of bacteria showed resistance to more than one antibiotic out of the eight different antibiotics used. Generally, out of all the isolates tested, 30.5% (29/95) showed resistance to more than one of the antibiotics tested. Both coagulase-negative and coagulase-positive* Staphylococcus* showed resistance to multiple drugs.

This was also reported in other studies [42, 43]. Bhatt et al. [44] reported isolation of high proportion of multidrug-resistant* Staphylococcus* organisms from surgical wounds. This presents a great threat as these organisms are commonly associated with a good number of animal and human infections. Bhatt et al. [44] also reported 56.4% multidrug resistance in Gram-positive bacteria and 66.9% multidrug resistance in Gram-negative bacteria. In another study, Raza et al. [45] reported multidrug resistance in Gram-positive and Gram-negative organisms to be at 47.5% and 88.33%, respectively. Expression of multiple drug resistance indicates that resistant organisms are developing mechanisms to counter effects of different antibiotics that are in use for treatment of bacterial infections.

The fact that some of the isolates showed some resistance to the antibiotics used, in this study, is important as it shows that normal flora/resident bacteria can harbor resistance genes to antibiotic(s). Transfer of resistance in bacteria has been documented to occur between different animal species, within humans, from animals to humans, and from humans to animals [46]. This makes it worth noting that transfer of resistance genes can occur to otherwise susceptible pathogenic bacteria, making them difficult to treat, not to mention transfer of resistance to human pathogenic bacteria. Some of these bacteria (30.5%) demonstrated multidrug resistance, thus worsening the situation. This study also identified antibiotics that can currently be used for treating respiratory or other infections in camels; Gentamicin and Tetracycline were found to be the most effective antibiotics. This will in turn enhance the health and productivity of Kenyan camels, leading to improvement of livelihood of the pastoral communities, as these animals act as their main source of income.

## Figures and Tables

**Table 1:** Bacterial isolation rates from the three counties.

Bacteria	Samburu *N* = 164		Isiolo *N* = 107		Nakuru *N* = 133	
	Number of isolated bacteria	% of isolates	Number of isolated bacteria	% of isolates	Number of isolated bacteria	% of isolates
*Bacillus*	67	40.85	60	56.07	33	24.81
	48	29.27	24	22.43	49	36.84
Coagulase-positive *Staphylococcus*	10	6.1	2	1.87	4	3
*Streptococcus agalactiae*	3	1.83	0	0	0	0
Other streptococci	36	21.95	21	19.63	47	35.34
